# Factors associated with grazing behavior in candidates for bariatric surgery at a hospital in the Amazon

**DOI:** 10.3389/fpubh.2023.1227214

**Published:** 2023-12-18

**Authors:** Jeane Lorena Dias Kikuchi, Manuela Maria de Lima Carvalhal, Mariana Maués de Castro, Vanessa Vieira Lourenço-Costa, Carlos Armando Ribeiro dos Santos, Flávia Siqueira Cunha, Carla Cristina Paiva Paracampo, Daniela Lopes Gomes

**Affiliations:** ^1^Postgraduate Program in Neurosciences and Behavior, Center for Theory and Research of Behavior, Federal University of Pará, Belém, Brazil; ^2^Faculty of Nutrition, Federal University of Pará, Belém, Brazil; ^3^Hospital Jean Bitar, Federal University of Pará, Belém, Brazil

**Keywords:** obesity, bariatric surgery, eating behavior, grazing, emotional eating

## Abstract

**Blackground:**

To assess grazing behavior and associated factors in candidates for bariatric surgery monitored at a public hospital that is a reference in the care of people with severe obesity.

**Methods:**

Cross-sectional analytical study, with candidates for bariatric surgery of both genders, treated in a public hospital in the Amazon. To assess grazing behavior, the Repetitive Eating Questionnaire was used, and to investigate patterns of eating behavior, the Three Factor Eating Questionnaire was used, which assesses: Emotional Eating, Cognitive Restriction and Uncontrolled Eating. Sociodemographic information was obtained through self-report and the description of medication use through the medical record. Body mass index (BMI) was also calculated by measuring weight and height. The SPSS program, v. 21.0 was used. The study was approved by the Research Ethics Committee.

**Results:**

A total of 205 participants were evaluated, with a mean age of 37.5 ± 8.6 years, the majority (93.7%) being women and the majority (59.5%) was not also using medication to lose weight. About 66.3% of the participants had compulsive grazing. The factor with the highest score was cognitive restriction (*p* < 0.001). Individuals who used weight loss drugs had higher scores in the cognitive restriction factor (*p* = 0.015) and lower scores for uncontrolled eating (*p* = 0.008), compulsive grazing (*p* = 0.021) and non-compulsive grazing (*p* = 0.034).

**Conclusion:**

Linear regression showed that emotional eating and uncontrolled eating were predictors of both compulsive grazing and non-compulsive grazing behavior. It was observed that grazing behavior, cognitive restriction, emotional eating and uncontrolled eating are present and correlated in the studied patients. In addition, the use of weight loss drugs seems to help reduce dysfunctional eating behaviors in patients with severe obesity.

## 1 Introduction

The cases of obesity have had a great growth in the last decades, being considered, currently, one of the biggest public health problems worldwide. Obesity is associated with chronic diseases such as cardiovascular disease, type 2 diabetes mellitus, osteoarthritis, obstructive sleep apnea and various types of cancer, thus contributing to a decrease in quality and life expectancy. In addition, it is also associated with social disadvantages and reduced socioeconomic productivity (1, 2).

Regarding the treatment for obesity, it is a complex and long-term treatment and involves lifestyle modification, consisting of a healthy diet, physical activity and medication use; it is essential. However, bariatric surgery has been the most effective option in cases where conventional clinical treatment does not have the expected effect, not only bringing more sustained and lasting weight loss, but also reducing the burden of comorbidities and the risk of mortality (3–5).

Obese patients and in the preoperative period of bariatric surgery may present harmful eating behaviors, such as “nibbling food,” emotional eating, binge eating and grazing, which make it difficult to control weight and can be an obstacle to treatment results, being related to worse results of bariatric surgery, especially in the long term (6–8).

Grazing behavior is defined as repeatedly eating small amounts of food in an unplanned way, in response or not to hunger or satiety, and can be divided into two subtypes: the compulsive, which is characterized by the perception that the individual is not able to resist eating and nibbles on food even in the absence of hunger; and the non-compulsive subtype, which is characterized by distracted eating several times (9, 10).

According to Kofman et al. (11), the grazing behavior is a behavioral phenomenon with great emphasis, it is highlighted that 46.6% individuals can be affected, being considered an emerging behavior nowadays. Therefore, it is noticed that dysfunctional eating behaviors, in particular the grazing behavior, are very common in candidates for bariatric surgery and can hinder the treatment for obesity.

Recently, researchers have investigated the presence of grazing behavior in obese individuals undergoing weight loss treatment, as in the research by Conceição et al. (6), who found that preoperative patients had a pattern of frequent grazing behavior, which was associated with worse clinical outcomes, since it made it possible to trigger overeating and, consequently, weight regain. Another study to be highlighted is that of Spirou et al. (12) who evaluated the grazing behavior in a sample with obesity and found significantly high scores in this population, concluding that individuals with obesity present a high frequency of grazing.

Despite the great relevance of the subject, there are still few studies that evaluate the presence of grazing during the preoperative period of bariatric surgery, mainly in Brazil. In this context, this study is justified by the fact that the topic is still recent and little explored, given the limited information on possible repercussions or sociodemographic, clinical, nutritional and behavioral factors related to grazing, especially in patients who are candidates for bariatric surgery. Our hypothesis is that some sociodemographic, clinical and nutritional characteristics and certain patterns of eating behavior are predictors of the development of grazing in these individuals.

In this context, due to the exponential growth of obesity and bariatric surgeries in Brazil, the objective of this study was to evaluate the characteristics of the grazing behavior and associated factors in patients who are candidates for bariatric surgery, monitored at a public hospital that is a reference in assisting people with severe obesity in Pará, Brazil.

## 2 Materials and methods

### 2.1 Study type

This is a cross-sectional analytical study, carried out in a public hospital in Belém, in the Amazon region of Brazil. The present study was approved by the Ethics Committee in Research with human beings from the Nucleus of Tropical Medicine of the Federal University of Pará (Opinion number 5.180.990), complying with the legal requirements in accordance with Resolution 466/2012, of the National Health Council, and in accordance with the Declaration of Helsinki. All participants signed the Free and Informed Consent Form (FICF).

### 2.2 Participants

A sample calculation was performed based on the number of patients seen in the month at the endocrinology service. Participated in the study individuals of both genders, aged 18 to 64 years, corresponding to the classification of adults, according to the World Health Organization (13), candidates for bariatric surgery, residents of the state of Pará, monitored at the endocrinology outpatient clinic of the Jean Bitar Hospital and who agreed to participate in the research by signing the FICF. Literate individuals who did not have diagnosed psychiatric disorders that compromised comprehension and writing were included. Those under 18 years old and over 64 years old, those who had diseases that interfered with body weight or eating behavior, people with a diagnosed eating disorder or those who did not respond to all questionnaires were excluded.

### 2.3 Place

Data capture and collection took place at the Endocrinology outpatient clinic of the Jean Bittar Hospital (HJB), in the city of Belém-Pará, which is the reference hospital unit in the State in the assistance to patients with obesity. HJB is one of the places where the “Zero Obesity” Program of the government of Pará, launched on September 10, 2020, was implemented, being the hospital that most performs bariatric surgeries by SUS in the Amazon. Patients are monitored by a multidisciplinary team composed of a surgeon, endocrinologist, nutritionist, psychologist and social worker both preoperatively and postoperatively, up to 2 years after surgery. However, after 2 years of the procedure, patients are referred to basic health units in order to be monitored by health professionals, except in the specialty of endocrinology, which continues to be monitored at the HJB.

### 2.4 Data collection and instruments

Data collection was carried out from February to September 2022. Participants were approached in the waiting room of the endocrinology sector while waiting for the medical consultation, where they were invited to participate in the research. After agreeing to participate in the study, the sociodemographic questionnaire was applied, then the weight and height were measured and later, the Repetitive Eating questionnaires REP (EAT)-Q and the Three Factor Eating Questionnaire (TFEQ-21) were applied, over time lasting 20 min in total. A total of 205 participants were evaluated, corresponding to 100% of the estimated sample. Non-probabilistic convenience sampling was performed. The Repetitive Eating questionnaire REP (EAT)-Q was used to assess grazing behavior, defined as repeatedly eating small amounts of food in an unplanned manner, as proposed by Conceição et al. (10). It is a questionnaire composed of 12 items, whose objective is to track the two subtypes of grazing behavior proposed by the authors: compulsive and non-compulsive. The participant answered each question using a Likert scale ranging from 0 (never) to 6 (every day), where each question is divided into subscales, questions 5–6–7-8-11-12 analyze compulsive grazing and questions 1–2–3-4-9-10 analyze non-compulsive grazing. The classification of having or not the behavior occurs through the average of points obtained by the total scale, in which the highest score is 6 points and the cutoff point is greater than or equal to 1.25, therefore, the person is considered with the grazing behavior if you obtain a score greater than or equal to 1.25.

To assess patterns of eating behavior, the Three Factor Eating Questionnaire, the TFEQ-21 short version, adapted by Tholin et al. (14) and translated and validated by Natacci and Júnior (15) in the Brazilian population was used. This instrument is composed of 21 Likert scale questions and investigates three behavioral factors of eating: emotional eating, cognitive restriction and uncontrolled eating. Emotional eating consists of 6 questions and is characterized by eating in response to an emotional state, that is, about the individual presenting changes in food intake due to mood alterations or challenging situations. Cognitive restriction is also composed of 6 questions that address obligations, prohibitions and food restrictions in order to maintain or lose weight, when food intake is restricted in order to lose weight. Uncontrolled eating consists of 9 questions and is defined by the presence of episodes of loss of self-control and excessive consumption of food in the presence or absence of hunger or when exposed to an external stimulus. The questions have four options: 1 – totally false; 2 – false most of the time; 3 – true most of the time; 4 – totally true. The higher the score, the greater the presence of the assessed behavior.

Information was obtained on sociodemographic characteristics, such as age, income in minimum wages, level of education (considering the last grade completed with approval), marital status (with or without a partner), origin (capital city or countryside), occupational situation, in addition to of information regarding the use of medication for weight loss prescribed by the endocrinologist and confirmed in the medical records of the participants. Weight loss drugs were those with a mechanism of action in the digestive system that prevent the absorption of ingested fat; those being active in the central nervous system regulating hunger and satiety (appetite suppressant); and those with peripheral action, with a blood glucose reduction mechanism.

Anthropometric measurements of weight and height were taken using a scale and a stadiometer in order to obtain body mass and height. The diagnosis of nutritional status was made by classifying the Body Mass Index (BMI) (16) to adults (≥ 20 years old and < 60 years old): Underweight (BMI < 18.5 kg/m^2^), Eutrophic (BMI ≥ 18.5 and < 25 kg/m^2^), Overweight (BMI ≥ 25 and < 30 kg/m^2^) and Obesity (BMI ≥ 30 kg/m^2^).

### 2.5 Data analysis

Microsoft Office Excel 2010 software was used for data tabulation. For statistical analysis, the Statistical Package for the Social Sciences (SPSS) software, version 21.0, was used. In descriptive statistics, data were expressed using measures of central tendency and dispersion. Statistical tests were chosen according to the classification of variables and sample distribution, which was evaluated using the Kolmogorov–Smirnov normality test, where the normality of the sample was found. Therefore, Spearman’s correlation test was used, a non-parametric test used to assess the relationship between two variables. From this test, the variables that showed statistically significant correlation were inserted into the multiple linear regression model to be used as a predictor model and thus model and predict relationships between variables. Having as dependent variables Compulsive grazing and co-variables: cognitive restriction, emotional eating, uncontrolled eating, gender and weight loss drugs use and Dependent variable: non-compulsive grazing; covariables: cognitive restriction, emotional eating, uncontrolled eating, gender and weight loss drug use. For comparison between the groups, the Mann–Whitney test was performed. In this test, the sample was divided into use and non-use of medication to lose weight. The level of statistical significance considered was *p* < 0.05.

## 3 Results

A total of 205 participants with a mean age of 37.5 ± 8.6 years were evaluated, of which 93.7% (*n* = 192; *p* < 0.001) were female, 35% of participants had completed high school (*n* = 72; *p* < 0.001), lived without a partner (*n* = 105; 51.2%; *p* < 0.001), had income >1 to 3 minimum wages (*n* = 96; 46.8%; *p* < 0.001) and resided in the state capital of Pará (n = 126; 61.5%; *p* < 0.001), in addition, most participants worked autonomously (*n* = 95;46.8%; *p* < 0.001). Regarding the use of weight loss drugs, 59.5% (*n* = 122; *p* = 0.008) did not use them. The mean BMI was 45.3 ± 6.7 kg/m2, and most participants had grade III obesity (*n* = 160; 78%; *p* = 0.063).

Regarding the grazing behavior, a higher frequency of compulsive grazing was observed 1.9 ± 1.2 compared to non-compulsive grazing 1.8 ± 1.3 (*p* = 0.007), with 56.6% (*n* = 116) of participants presented non-compulsive grazing and 66.3% (*n* = 136) presented compulsive grazing. From the TFEQ-21, it was found that the cognitive restriction factor was the most frequent in the participants (52.7 ± 20.0), followed by the emotional eating factor (41.2 ± 30.3) (*p* < 0.001) (Table 1).

**Table 1:** Sociodemographic, anthropometric and eating behavior characterization of candidates for bariatric surgery monitored at a reference public hospital, Belém-PA.

	*n* / Mean± SD^a^	Interval / %	Value of *p*^b^
Age	37.5 ± 8.6	20–60	
Gender			
Female	192	93.7	<0.001
Male	13	6.3	
Education			
Incomplete middle school	10	4.9	<0.001
Complete middle school	11	5.4	
Incomplete high school	19	9.3	
Complete high school	72	35.1	
Incomplete higher education	40	19.5	
Complete higher education	53	25.9	
Marital status			
With a partner	100	48.8	<0.001
Without a partner	105	51.2	
Income (minimum wages)^a^			
Unemployed	6	2.9	<0.001
< 1 minimum wage	15	7.3	
1 minimum wage	31	15.1	
> 1–3 minimum wages	96	46.8	
>3–5 minimum wages	40	19.5	
> 5 minimum wages	17	8.3	
Origin			
Capital	126	61.5	0.001
Countryside	79	38.5	
Weight loss drugs			
Yes	83	40.5	0.008
No	122	59.5	
Occupational situation			
Unemployed	1	5	<0.001
Formal employment	61	28.8	
Freelance	96	46.8	
Works at home	37	18	
Student	9	4.4	
Retired	1	5	
Body mass index (kg/m^2^)	45.3 ± 6.7	31.2–74.5	
Grade I Obesity	5	2.4	0.063
Grade II Obesity	40	19.5	
Grade III Obesity	160	78	
Current weight (kg)	117.9 ± 20.8	80.4–201.0	
Expected weight after surgery (kg)	68.23 ± 10.4	40.0–100.0	
Non-compulsive grazing	1.8 ± 1.3	0.0–5.8	-
Without	89	43.4	0.069*
With	116	56.6	
Compulsive grazing	1.9 ± 1.2	0.0–5.8	-
Without	69	33.7	<0.001*
With	136	66.3	
Dietary pattern			
Cognitive restriction	52.7 ± 20.0	5.6–100.0	<0.001**
Emotional eating	41.2 ± 30.3	0.0–100.0	
Uncontrolled eating	37.9 ± 21.0	0.0–100.0	

^a^SD = Standard deviation; ^b^Qui-square; minimum wage = $BRL 1.212; *Binomial test; **Friedman test.

No correlation was found between weight or BMI and different aspects of eating behavior. However, there was a statistically significant negative correlation between the cognitive restriction factor and the factors of emotional eating (*r*^2^ = −0.334; *p* < 0.001), uncontrolled eating (*r*^2^ = −0.383; *p* < 0.001), non-compulsive grazing behavior (*r*^2^ = 0.296; *p* < 0.001) and compulsive grazing behavior (*r*^2^ = −0.305; *p* < 0.001). Emotional eating was positively correlated with non-compulsive grazing (*r*^2^ = 0.563; *p* < 0.001) and compulsive grazing (*r*^2^ = 0.647; *p* < 0.001). Considering uncontrolled eating, there was a positive correlation with non-compulsive grazing behavior (*r*^2^ = 0.537; *p* < 0.001), compulsive grazing (*r*^2^ = 0.587; *p* < 0.001) and emotional eating factor (*r*^2^ = 0.640; *p* < 0.001). It was possible to verify a negative correlation between age with emotional eating factor (*r*^2^ = −0.142; *p* = 0.021), the cognitive restriction factor (*r*^2^ = 0.209; *p* = 0.001), BMI (*r*^2^ = −0.173; *p* = 0.007), compulsive grazing (*r*^2^ = −0.161; *p* = 0.010) and non-compulsive grazing (*r*^2^ = −0.119; *p* = 0.045) (Table 2).

**Table 2:** Bivariate correlation analysis between different patterns of eating behavior, the presence of grazing, age and nutritional status in candidates for bariatric surgery monitored at a reference public hospital, Belém-PA.

	*r*^2^	Value of p*
Cognitive restriction		
Emotional eating	−0.334	**<0.001**
Uncontrolled eating	−0.383	**<0.001**
Non-compulsive grazing	−0.296	**<0.001**
Compulsive grazing	−0.305	**<0.001**
Emotional eating	*r*^2^	Value of p*
Non-compulsive grazing	0.563	**<0.001**
Compulsive grazing	0.647	**<0.001**
Uncontrolled eating	*r*^2^	Value of p*
Non-compulsive grazing	0.537	**<0.001**
Compulsive grazing	0.587	**<0.001**
Emotional eating	0,640	**<0.001**
Age	*r*^2^	Value of p*
Uncontrolled eating	−0.024	0.365
Emotional eating	−0.142	0.021
Cognitive restriction	0.209	0.001
BMI	−0.173	0.007
Compulsive grazing	−0.161	0.010
Non-compulsive grazing	−0.119	0.045
BMI	*r*^2^	Value of p*
Cognitive restriction	−0.047	0.252
Emotional eating	−0.066	0174
Uncontrolled eating	−0.017	0.406

Spearman’s correlation test, statistical significance *p* < 0.05. Bold values in statistical significance.

It was observed that individuals who used weight loss drugs had higher scores in the cognitive restriction factor (*p* = 0.015) and lower scores in the uncontrolled eating factor (*p* = 0.008), in addition, they also had statistically lower scores for compulsive grazing (*p* = 0.021) and non-compulsive grazing (*p* = 0.034) (Table 3).

**Table 3:** Eating behavior according to the use of weight loss drugs in candidates for bariatric surgery monitored at a reference public hospital, Belém-PA.

Eating behavior	Weight loss drugs use				Value of p*
	Yes (*n* = 83)		No (*n* = 122)		
	Mean ± DP	Median (P5–P95)	Mean ± DP	Median (P5–P95)	
Cognitive restriction	56.6 ± 19.1	55.6 (11.1–94.4)	50.0 ± 20.3	50.0 (5.6–100.0)	0.015
Emotional eating	37.5.5 ± 27.2	33.3 (0.0–100)	43.7 ± 32.1	38.9 (0.0–100.0)	0.230
Uncontrolled eating	33.3 ± 19.2	29.6- (3.7–96.3)	41.0 ± 21.7	38.9 (0.0–100.0)	0.008
Compulsive grazing	1.7 ± 1.1	1.5 (0.0–5.8)	2.1 ± 1.3	1.8 (0.2–5.8)	0.021
Non-compulsive grazing	1.5 ± 1.0	1.2- (0.0–4.8)	2.0 ± 1.4	1.6 (0.0–5.8)	0.034

*Mann–Whitney test.

According to the statistical significance indicated in the bivariate analysis, variables were chosen for the models in the multiple linear regression. Table 4 shows the correlation between compulsive grazing behavior and emotional eating (*β* =0.452; CI = 0.013; 0.024; *p* < 0.001) and uncontrolled eating (*β* = 0.285; CI = 0.009; 0.026; *p* < 0.001), which remained statistically significant regardless of gender and use of weight loss medication (Table 4).

**Table 4:** Multiple linear regression between compulsive grazing behavior and the factors of emotional eating and uncontrolled eating in candidates for bariatric surgery monitored at a reference public hospital, Belém-PA.

Compulsive grazing	*B*	CI 95% (minimum; maximum)	Value of *p*
Model 1			
Cognitive restriction	−0.036	−0.009-0.005	0.517
Emotional eating	0.452	0.013–0.024	**<0.001**
Uncontrolled eating	0.285	0.009–0.026	**<0.001**
Model 2			
Cognitive restriction	−0.029	−0.009-0.005	0.605
Emotional eating	0.461	0.013–0.025	**<0.001**
Uncontrolled eating	0.285	0.009–0.025	**<0.001**
Gender	0.044	−0.305-0.754	0.405
Model 3			
Cognitive restriction	−0.023	−0.008-0.006	0.682
Emotional eating	0.464	0.014–0.025	**<0.001**
Uncontrolled eating	0.275	0.008–0.025	**<0.001**
Gender	0.043	−0.307-0.752	0.408
Weight loss drugs use	0.055	−0.122-0.403	0.292

Linear regression; Dependent variable: Compulsive grazing; co-variables: cognitive restriction, emotional eating, uncontrolled eating, gender and weight loss drugs use, B, regression coefficient. Bold values in statistical significance.

Table 5 shows the multiple linear regression analysis with the correlation between non-compulsive grazing behavior and the factors of emotional eating (*β* = 0.347; CI = 0.009; 0.022; *p* < 0.001) and uncontrolled eating (*β* = 0.306; CI = 0.010; 0.029; *p* < 0.001), which remained statistically significant regardless of gender and use of weight loss medication.

**Table 5:** Multiple Linear Regression between non-compulsive grazing behavior and the factors of emotional eating and uncontrolled eating in candidates for bariatric surgery monitored at a reference public hospital, Belém-PA.

Non-compulsive grazing	*B*	CI 95% (minimum; maximum)	Value of *p*
Model 1			
Cognitive restriction	−0.086	−0.014-0.002	0.151
Emotional eating	0.347	0.009–0.022	**<0.001**
Uncontrolled eating	0.306	0.010–0.029	**<0.001**
Model 2			
Cognitive restriction	−0.082	−0.013- 0.002	0.175
Emotional eating	0.352	0.009–0.022	**<0.001**
Uncontrolled eating	0.305	0.010–0.029	**<0.001**
Gender	0.022	−0.480-0.721	0.692
Model 3			
Cognitive restriction	−0.072	−0.13-0.003	0.231
Emotional eating	0.357	0.009–0.022	**<0.001**
Uncontrolled eating	0.290	0.009–0.028	**<0.001**
Gender	0.022	−0.480-0.716	0.698
Weight loss drugs use	0.089	−0.056-0.536	0.112

Linear regression; Dependent variable: non-compulsive grazing; co-variables: cognitive restriction, emotional eating, uncontrolled eating, gender and weight loss drugs use, B, Regression coefficient. Bold values in statistical significance.

## 4 Discussion

The present study sought to evaluate the characteristics of eating behavior and factors associated with grazing behavior in patients in the preoperative period of bariatric surgery, monitored at a public hospital that is a reference in the care of people with severe obesity in Pará, Brazil.

Regarding eating behavior, there was a difference between the prevalence of compulsive and non-compulsive grazing. It is important to note that both in patients with obesity undergoing clinical treatment and in candidates for bariatric surgery, grazing behavior can be found and is associated with worse results in weight loss and impairment of mental health (8).

A higher frequency of compulsive grazing was found in relation to non-compulsive grazing. Similarly, Walø-Syversen et al. (17), also using the Repetitive Eating Questionnaire REP (EAT)-Q, found a higher prevalence of compulsive grazing in their study sample, so this subtype seems to be more present in these patients.

To further reinforce this finding, Heriseanu, Hay and Touyz (18) concluded that susceptibility to compulsive grazing was increased by 11 times in individuals with obesity and this condition was associated with worse outcomes and greater impact on quality of life. One hypothesis to explain the significant prevalence of the compulsive grazing subtype would be that in this population there is still preservation of the gastric reservoir, which would make patients eat more and more frequently without feeling so much discomfort.

In our study, using the TFEQ-R21, we found that cognitive restriction was the most frequent factor in the participants, diverging from the study by Akkayaoğlu and Celik (19), who also used the TFEQ, and found that patients before bariatric surgery had a higher score in the uncontrolled eating factor. Cognitive restriction can be characterized as an intentional restriction to maintain or reduce weight. According to Cifuentes et al. (20) the cognitive restriction is more observed in patients with obesity and individuals who diet, and when used appropriately, it allows dieters to control their food intake.

Correlation analyzes showed that as emotional eating increases, compulsive and non-compulsive grazing behavior increases, thus demonstrating that this is a factor that worsens the pattern of eating behavior of the studied individuals. In multiple linear regression analysis, it was observed that compulsive grazing behavior was correlated with emotional eating and uncontrolled eating, regardless of gender and medication use, which suggests that this correlation seems not to be controlled through the use of weight loss drugs.

Heriseanu et al. (8) report that grazing behavior is very prevalent in obesity and eating disorders. There is evidence to indicate that grazing (in particular the “compulsive” subtype) is associated with worse weight loss treatment outcomes in obesity, increased risk of eating disorders, and mental health worsening.

Conceição et al. (10) found strong correlations between compulsive grazing behavior and eating disorder symptoms, as well as in the study by Heriseanu, Hay and Touyz (2019) (18) in which participants in the group that scored higher on the compulsive grazing subtype showed stronger associations with characteristics of eating disorders, in particular with binge eating, than participants in the non-compulsive grazing group.

Similar to our findings, the studies by Aymes et al. (21) also found correlations between emotional eating and uncontrolled eating; in addition to Lourdes et al. (22) who observed an increase in the frequency of compulsive and non-compulsive grazing with emotional eating and uncontrolled eating, both in patients with severe obesity undergoing weight loss treatment.

Emotional eating is related to food intake not due to physical hunger, but motivated by triggering factors such as feelings, mood and emotions, especially aversive ones (15). The literature points to emotional eating as a behavior that negatively affects weight maintenance in the postoperative period, therefore, it is necessary to direct strategies that reduce these dysfunctional eating behaviors from the preoperative monitoring (23) Given this, our hypothesis is that people with obesity can often act guided by aversive emotions and emit problematic eating behaviors, which highlights the role of grazing behavior in obesity.

It was also found that non-compulsive grazing behavior was also correlated with emotional eating and uncontrolled eating, and remained regardless of gender and medication use. Conceição et al. (6) found that the non-compulsive grazing subscale was correlated only with uncontrolled eating and emotional eating. Additionally, our finding points out that the use of weight loss drugs does not seem to be enough to change the tendency toward increased emotional eating and uncontrolled eating in people with non-compulsive grazing.

It should be noted that, although the above studies did not differentiate between genders and did not include the use of medications as in our study, the literature already suggests that 20–60% of candidates for bariatric surgery may have some type of grazing behavior, indicating that this is a common behavior in this population (8, 12) corroborating our findings.

A negative correlation was found between age, emotional eating, cognitive eating, BMI and grazing subtypes. Costa et al. (24), when evaluating the nibbling behavior in patients with severe obesity referred to bariatric surgery through a specific questionnaire on eating behavior with questions about “nibbling food,” found that younger individuals (under 35 years old) had a higher frequency of this type of behavior. Abdella et al. (25) investigated the relationships between eating behaviors, the influence of age (≤25 years group and > 25 years group), sex, BMI and obesity-associated genotype (FTO) on these relationships. They found an inverse relationship between age lack of eating control and emotional eating for the entire sample, in addition to a decline in emotional eating with increasing age, especially in the FTO AA + AT genotype group, however, unlike our study, they found a correlation positive between age and BMI. Therefore that age can influence the patients eating behavior.

Individuals who used weight loss drugs had higher scores in the cognitive restriction factor and lower scores in the uncontrolled eating factor and in compulsive and non-compulsive grazing behaviors, which suggests that the use of these drugs may have a positive influence on dysfunctional eating behavior.

Corroborating the above, Tham et al. (26) when testing the use of drugs for the treatment of obesity in adults with risk factors for metabolic syndrome and with acute myocardial infarction in an obesity control clinic, they were able to notice weight loss, BMI reduction and reduction in waist circumference after using these medications. An improvement in glycemic control, hypercholesterolemia and rates of systemic arterial hypertension was also found, as well as a significant reduction in levels of depression, anxiety and stress, in addition to improvements in eating behavior patterns with the use of weight loss drugs.

Safer et al. (27), in a randomized clinical trial, found that the combination of phentermine and topiramate (generally used in the treatment of obesity) in patients with binge eating disorder (BED), in addition to having acted to reduce the total frequency of BED episodes, significantly promoted the BMI decrease. The above investigations that evaluate eating behavior and the use of medication reinforce the importance of the findings of our study, thus showing that the use of medication to lose weight seems to be a good mechanism for improving the eating behavior of patients with obesity. Is it possible that patients using weight loss therapies are more aware to the obesity problem because they sought/accepted treatment.

The present study has limitations, such as the collection of sociodemographic information through self-reports. However, this study may be a support for clinical practice, and may, from then on, guide future intervention studies. In addition, it provides information on the prevalence of grazing behavior in candidates for bariatric surgery and on the relationship between the use of weight loss drugs and different patterns of eating behavior, which until then have been little studied. It is suggested that new multicentric studies be carried out with representative samples, as well as follow-up studies, comparing the prevalence of eating behavior patterns in the preoperative period and at different moments in the postoperative period of bariatric surgery.

## 5 Conclusion

In this study, it was observed that most participants had compulsive grazing and cognitive restriction behavior. In addition, compulsive and non-compulsive grazing behavior was associated with emotional eating and uncontrolled eating, regardless of gender and use of weight loss medications. It was found that participants who used weight loss drugs had higher levels of cognitive restriction and a lower frequency of compulsive and non-compulsive grazing behavior, indicating that the use of weight loss drugs seems to help reduce dysfunctional eating behaviors in patients with severe obesity.

The findings of this study point to the importance of identifying dysfunctional behaviors before bariatric surgery in order to intervene early and prevent postoperative complications due to patterns of disordered eating behavior that were maintained or even recovered long after surgery. It is suggested that modeling dysfunctional eating behaviors may help in adherence to treatment and decrease the risk of failures in weight control in the long term.

## Data availability statement

The raw data supporting the conclusions of this article will be made available by the authors, without undue reservation.

## Ethics statement

The studies involving humans were approved by the Ethics Committee in Research with human beings from the Nucleus of Tropical Medicine of the Federal University of Pará (Opinion N. 5,180,990), complying with the legal requirements in accordance with Resolution 466 of December 12, 2012, of the National Health Council, and in accordance with the Declaration of Helsinki. All participants signed the Free and Informed Consent Form (FICF). The studies were conducted in accordance with the local legislation and institutional requirements. The participants provided their written informed consent to participate in this study.

## Author contributions

JK: protocol, project development, manuscript writing, editing, review & editing and performed the statistical analysis. ML: protocol, project development, manuscript writing, editing, and review & editing. MC: organized the database, manuscript writing, and editing. VV: protocol, project development, manuscript writing, and editing. CS: protocol, project development, manuscript writing, and editing. FC: protocol, project development, and manuscript writing. CP: supervision, conceptualization, data collection or management, data analysis, performed the statistical analysis, manuscript writing, and editing. DG: supervision, conceptualization, data collection or management, data analysis, manuscript writing, and editing. All authors contributed to the manuscript revision, read, and approved the submitted version.
